# Reply to Huang et al.: Debunking the Sangdanlin myth

**DOI:** 10.1073/pnas.2403014121

**Published:** 2024-04-29

**Authors:** Jun Meng, Stuart A. Gilder, Xiaodong Tan, Xin Li, Yalin Li, Hui Luo, Noritoshi Suzuki, Zihao Wang, Yuchen Chi, Chunyang Zhang, Chengshan Wang

**Affiliations:** ^a^State Key Laboratory of Biogeology and Environmental Geology, School of Earth Sciences and Resources, China University of Geosciences Beijing, Beijing 100083, China; ^b^Department of Earth and Environmental Sciences, Ludwig Maximilians University, Munich 80333, Germany; ^c^Key Laboratory of Marginal Sea Geology, South China Sea Institute of Oceanology, Chinese Academy of Sciences, Guangzhou 510301, China; ^d^State Key Laboratory of Palaeobiology and Stratigraphy, Nanjing Institute of Geology and Palaeontology and Center for Excellence in Life and Paleoenvironment, Chinese Academy of Sciences, Nanjing 210008, China; ^e^Department of Earth Science, Graduate School of Science, Tohoku University, Sendai City 980-8578, Japan

The size of Greater India has been a long-standing mystery for a century and bears significantly on our understanding of the India–Asia collision. The Sangdanlin section in southern Tibet represents a geologic Rosetta stone to reconcile Greater India controversies. Meng et al. (1) presented paleogeographic reconstructions stemming from paleomagnetic data and a revised age model of the Sangdanlin section, which together demonstrated Greater India extended ~3,000 km as a single plate before subducting under Asia.

Huang et al. (2) call into question our revised age model citing undocumented field observations. Our study was not the first to find chaotic intermixing in southern Tibet as already discussed (1); we stand by our published field observations. Huang et al. (2) do not question our paleomagnetic data from Sangdanlin, which is consistent with the finding of Yuan et al. (3), except that Yuan et al. (3) thought the strata they sampled for paleomagnetism was mid-Paleocene and we show it is Early Cretaceous. Yuan et al.’s (3) age assignment was based on the radiolarian taxonomy by Hu et al. (4) for unit 6 of the Sangdanlin section. Huang et al. (2) provide no new data to support their claim that unit 6 is Paleocene nor why they consider our Cretaceous radiolaria to be reworked.

First, we identified radiolaria from scanning electron microscope (SEM) images, which is widely considered as the most robust method (5). Ranges of all species within one sample yielded a consistent age assignment, with no mixing of species from different ages. Unit 6 has solely a Barremian to Aptian primary radiolaria assemblage. Besides unit 6, we found Early Cretaceous radiolaria in units 9 and 17. Together with Wang (6), 4,700 SEM photos went into our analysis allowing us to conclusively assign unit 6 as Early Cretaceous, not Paleocene. Both the Early Cretaceous radiolaria in units 6, 9, and 17 and the Paleocene radiolaria in units 11 (upper), 13, 15, and 20 together argue that the entire section has an incoherent stratigraphy, with a complicated tectonic repetition. Moreover, we showed the strata of the two ages are both magnetically and geochemically distinct.

Second, we neither ignored Hu et al. (4) nor Ding (7), both were cited in our paper. Conversely, we carefully reviewed all published paleontological work at Sangdanlin—that includes the erroneous arguments of Huang et al. (2) based on the work of Hu et al. (4), who identified radiolaria from transmitted light microscope-based observations, not from SEM images. Light microscope observations cannot distinguish segmentation patterns in radiolaria, the detailed cephalic (top of the cone) structure, nor the surface ornamentation, all of which are needed to identify species, genera, or family. The unreliability of their method has been shown since the 1940s (5). Not only us—neither Chan (8) nor Wang (6) found Paleocene and Cretaceous radiolaria mixed together. Unless Huang et al. (2) produce data to the contrary, their claims on reworked radiolaria are unsubstantiated. The rest of the comment concerns pole selection, which we discussed in other publications (9, 10). This subject cannot be treated simply in a comment or in a reply to a comment.
